# Does Increased Femoral Anteversion Can Cause Hip Abductor Muscle Weakness?

**DOI:** 10.3390/children10050782

**Published:** 2023-04-26

**Authors:** Adnan Apti, Nazif Ekin Akalan

**Affiliations:** Faculty of Health Sciences, Division of Physiotherapy and Rehabilitation, Istanbul Kültür University, Istanbul 34158, Turkey

**Keywords:** femoral anteversion, hip abductor muscle strength, hip kinematics, walking, children

## Abstract

Background: Increased femoral anteversion (IFA) causes functional problems (i.e., tripping, frequently falling, and fatigue) by affecting the pelvis and lower extremity biomechanics. In the frontal plane, increased contralateral pelvic drop and ipsilateral hip adduction, which are mainly considered deteriorated hip abductor muscle mechanisms, are associated with hip and knee injuries. Aims: The aim of this study was to examine the effects of femoral anteversion on hip abductor weakness and frontal plane pelvis–hip biomechanics during walking. Methods: The study included nine subjects with increased femoral anteversion and a control group of eleven subjects. Maximum isometric voluntary contraction (MIVC) values of the hip abductor muscles were measured with a handheld dynamometer. Three-dimensional gait analysis was performed for kinetic, kinematic, and temporo-spatial gait parameters. Non-parametric tests were used for statistical analysis (*p* < 0.05). Results: There was no significant difference found between the MIVC values of the IFA and control groups (*p* = 0.14). Moreover, no significant difference was determined between the ipsilateral peak hip adduction (*p* = 0.088) and contralateral pelvic drop (*p* = 0.149) in the stance phase. Additionally, there was no correlation between the peak hip adduction angle in the stance phase and normalized MIVC values in the IFA group (r = −0.198, *p* = 0.44), or in the control group (r = −0.174, *p* = 0.55). The deviations of pelvic rotation (*p* = 0.022), hip internal rotation (*p* = 0.003), and internal foot progression (*p* = 0.022), were found to be higher in the IFA group than in the controls. Conclusions: IFA may not be associated with hip abductor muscle weakness, and it may not lead to the hip adduction and pelvic depression that can be seen in hip abductor weakness. Increased pelvic rotation and internal hip rotation during walking might be considered as a compensation for the femoral head–acetabulum alignment mechanism in the frontal plane.

## 1. Introduction

**Figure 1:**
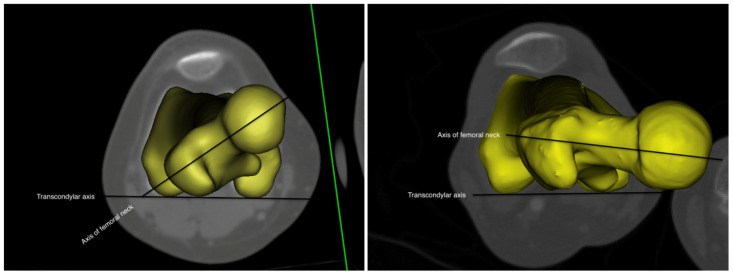
The left figure represents increased femoral anteversion, and the right figure femoral retroversion.

Increased femoral anteversion is referred as the anterior rotation of the femoral head in relation to the transcondylar axis of the knee. (Figure 1) [1]. The femoral anteversion angle decreases with age and reaches normal values until bone development is complete [2]. It was reported that increased femoral anteversion causes significant changes in daily functional activities, walking, and running, compared to controls [3,4,5,6]. Studies have indicated that increased femoral anteversion has a role in pain and musculoskeletal injuries through both static and dynamic overload mechanisms. Abnormal pelvis and hip joint mechanics can lead to overload forces on intra-articular and extra-articular hip and groin tissues [7,8,9]. Although it is known that derotation osteotomies are beneficial for individuals with a femoral anteversion increase of ≥50°, the information available in the literature is limited to the prevention or rehabilitation of problems related to anteversion increase at lower angles.

Limited research has indicated that increased femoral anteversion may lead to hip abductor muscle weakness, although conflicting conclusions have been reported [10,11,12]. Hip abductor weakness (i.e., gluteus medius) causes increased ipsilateral hip adduction during the stance phase of walking [13,14]. Studies examining walking and running have shown that greater hip adduction in stance contributes to certain musculoskeletal injuries such as patellofemoral pain [7,8,15]. Increased hip adduction in the stance phase of running is associated with increased femoral anteversion [16]. However, its relationship to direct anteversion or anteversion-induced hip abduction weakness is not well known. In addition, it is not clear whether increased femoral anteversion leads to hip abductor weakness and an increased hip adduction angle in walking.

The hypothesis of this study was that increased femoral anteversion alters the orientation of the greater trochanter, leading to hip abductor muscle weakness, which increases hip adduction during the stance phase of gait. The aim of this study was to investigate whether increased femoral anteversion influences the hip abductor muscle strength and frontal plane pelvis–hip biomechanics in children.

## 2. Materials and Methods

### 2.1. Study Design

This study was approved by Clinical Research Ethics Committee of the Bakırköy Dr. Sadi Konuk Training and Research Hospital on 25 August 2014 with approval number 2014/11/10. This prospective controlled study was conducted with the participation of volunteers aged 5–15 years old. To determine the effects of increased femoral anteversion on hip joint biomechanics, 9 participants with increased femoral anteversion (3 male and 6 female, mean age: 9.5 ± 2.97 years) and 11 healthy peers as a control group (7 male and 4 female, mean age: 10.25 ± 3.93 years) were included in this study. All assessments were performed in the Gait Analysis Laboratory of Istanbul University, Istanbul Medical Faculty, between March 2015 and March 2017. The study inclusion criteria were determined as follows: (i) no history of orthopedic surgery; (ii) no history of neurological surgery; (iii) absence of musculoskeletal disorders; and (iv) absence of generalized joint hypermobility. Written informed consent was obtained from participants and their parents. Prior to the tests, researchers were assured that the subjects had not engaged in any high-level sportive activities in the last 3 days. All the tests and measurements were performed by an experienced physiotherapist. The participants were divided into two groups: increased femoral anteversion and control. To exclude the effects of hypermobility, participants with a Beighton score of 5 and above were excluded from both groups [17].

### 2.2. Data Collection

**Figure 2:**
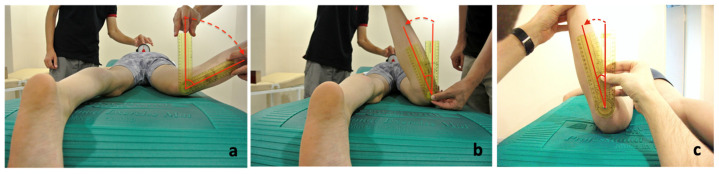
(**a**) Hip internal rotation, (**b**) hip external rotation, and (**c**) Craig’s test.

Hip rotation angles were measured in the prone position using a goniometer. Participants with a Craig’s test of ≥30 degrees and hip rotation of ≥60 degrees internal as well as ≥20 degrees external were included in the increased femoral anteversion group [18,19,20]. Craig’s test, also known as the trochanteric prominence test, is the determination of the hip internal rotation angle measured when the trochanter major is most prominent on the lateral surface of the hip [18]. For hip internal rotation measurement, the knee was flexed 90 degrees while the participant was lying comfortably in the prone position and the hips waited for 5–10 s to reach maximum internal rotation with the effect of gravity. The angle between the vertical axis and the tibial shaft was measured with a goniometer and recorded as the internal rotation angle. To determine the hip external rotation range of motion, it was measured at the maximum external rotation angle that could be performed without pelvic rotation. During the measurement, the neutral position of the pelvis was monitored with an inclinometer placed on both spina iliaca posterior superior (Figure 2). According to the measurements, the hip internal rotation must be at least 40 degrees greater than the hip external rotation for inclusion in the increased femoral anteversion group [21]. The control group subjects had lesser internal and greater external hip rotation than the increased femoral anteversion group. Both groups provide settled criteria in lower extremities bilaterally. Therefore, evaluated parameters were collected from both the left and right sides. For hip abductor muscle strength, maximal isometric voluntary contractions (MIVC) were measured with a handheld dynamometer (HHD, Microfet 2, Hoggan Health Industries, West Jordan, UT, USA) in a side-lying position [22,23]. Five repetitive MIVC measurements were taken with 2 min resting intervals for each side. The average of the three highest values was taken as the mean MIVC value. The mean MIVC values were normalized with respect to body weight, considering the age range (5–15 years) of the participants [23]. Standardized verbal statements were given to ensure maximum participation of the participants. Gait analysis was conducted to determine kinetic, kinematic, and temporal as well as spatial parameters using an optoelectronic system (ELITE 2002; BTS, Milan, Italy) and two force-plates (Kistler, MS, USA). Passive retroreflective markers were placed on the skin at specific anatomical points as described by Davis et al. [24]. Gait data were obtained at the participants’ self-selected walking speeds. All measurements were acquired at laboratory temperatures of 22–25 °C.

### 2.3. Statistical Analysis

Power analysis showed that the number of participants [18] included was above the minimum sample size required to ensure a power of 95% confidence level and to detect statistical significance at a two-sided significance level of 0.05 (*β* = 0.2) [25]. Data obtained in the study were analyzed statistically using IBM-SPSS vn. 24 software. The Shapiro–Wilk test and Kolmogorov–Smirnov test were used to determine the normality of the data. To calculate significant differences between the groups in respect of the relevant gait parameters, the Wilcoxon test was used. Correlations between data were examined with Spearman’s correlation test. A value of *p* < 0.05 was accepted as statistically significant.

## 3. Results

It was determined that there was no significant difference between the mean age of the participants in the increased femoral anteversion group (9.5 ± 2.97) and the mean age of the participants in the control group (10.25 ± 3.98) (*p* = 0.385). In addition, no statistically significant differences were found between the mean BMI values of the increased femoral anteversion group (18.49 ± 3.5) and the control group (18.10 ± 6.05) (0.461). The average of the maximum hip internal rotation angle is significantly higher in the increased femoral anteversion group (68.33 ± 6.34) than the control group (38.95 ± 13.66) (*p* = 0.001). Additionally, the average femoral anteversion angle as measured by Craig’s test is significantly higher in the increased femoral anteversion group (42.83 ± 10.29) compared to the control group (21.25 ± 12.25) (*p* = 0.001). In the IFA group, compared to the control group, the hip internal rotation angles and Craig’s test score were both significantly higher, while the hip external rotation angles were significantly lower (Table 1). Normalized hip abductor MIVC strength values were not statistically different in the IFA (4 ± 0.57 N/kg) and control (3 ± 0.89 N/kg) groups (*p* = 0.14, Table 1).

**Figure 3:**
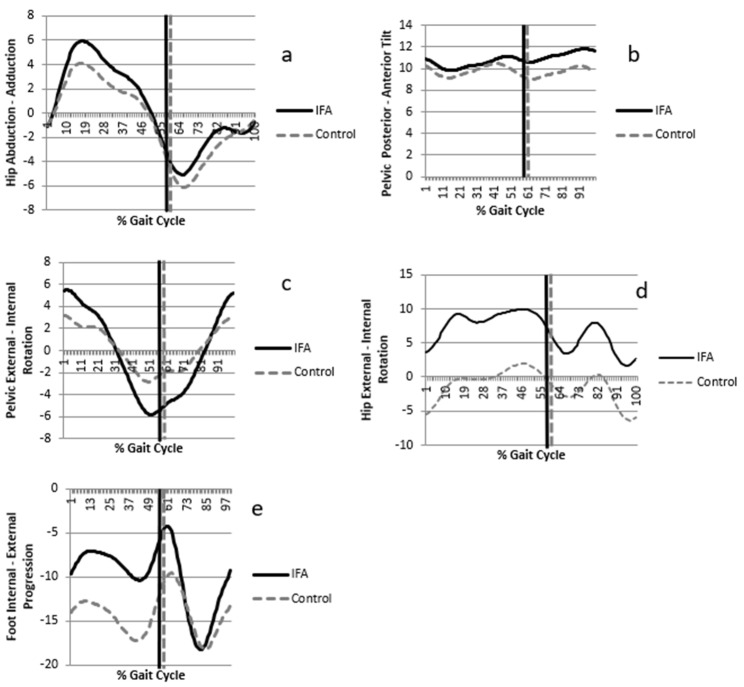
The bold line (IFA) and dashed line (control) represent the kinematics of the pelvis, hip, and foot on the sagittal, frontal, and transverse planes for all participants. IFA: increased femoral anteversion; (**a**) hip abduction–adduction, (**b**) pelvic posterior–anterior tilt, (**c**) pelvic rotation, (**d**) hip external–internal rotation, and (**e**) foot internal–external progression.

Gait parameters were obtained at the participants’ self-selected walking speed, which was found to be not significantly different between the groups (1.13 ± 0.13 m/s for the IFA group and 1.27 ± 0.11 m/s for the control group, *p* = 0.87). According to the kinetic analysis, it was determined that there was no significant difference between the groups in the hip abductor moment values in the stance phase of walking (IFA group: −0.32 ± 0.33, control group: 0.20 ± 0.15, *p* = 0.35). In the kinematic analysis, the peak range of motions was investigated for the pelvis and lower extremity joints in the stance (Table 2). The peak hip adduction angle in the stance was not statistically different between the IFA (6.12 ± 4.97) and control groups (4.34 ± 4.03) (*p* = 0.08, Figure 3a).

The range of motion of the pelvis in the frontal plane (pelvic obliquity) was found to be significantly higher in the IFA group (IFA: 9.67 ± 5.36) than in the control group (6.33 ± 2.55, *p* = 0.025). No significant decrease was determined in the contralateral peak pelvic depression in the stance (IFA: −4.25 ± 3.67, control group: −2.8 ± 1.92, *p* = 0.14). When the kinematics of the pelvis in the sagittal plane were examined, no significant difference was observed between the groups in the angle of the peak pelvic tilt (IFA: 12.82 ± 5.89, control group: 11.44 ± 2.78, *p* = 0.37) (Figure 3b).

In the transverse plane, the peak pelvic rotation (IFA: 14.1 ± 5.7, control group: 8.7 ± 4.5, *p* = 0.02) (Figure 3c), the peak hip internal rotation angle (IFA: 12.2 ± 7.3, control group: 3.8 ± 5.1, *p* = 0.003) (Figure 3d), and the peak foot internal rotation angle in the stance (IFA: −3.18 ± 7.29, control group: −9 ± 7.59, *p* = 0.022) (Figure 3e) were determined to be higher in the IFA group than in the control group (Table 2). Each significantly different parameter was above the minimum clinically important difference score, which was calculated as defined in the literature (10.9, 6.3, and −6.2, respectively) [26].

The relationship between hip abductor MIVC and increased hip adduction in the stance phase was assessed with Spearman’s correlation analysis, and no correlation was determined between peak hip adduction in the stance phase and normalized MIVC (N/kg) values in either the IFA group (r = −0.198, *p* = 0.44), or the control group (r = −0.174, *p* = 0.55). No correlation was found between the femoral anteversion angle (Craig’s test) and hip adduction in the stance phase (*r* = 0.149, *p* = 0.49).

## 4. Discussion

In this study, the effects of increased femoral anteversion on hip abductor muscle weakness, and whether there is an increase in the peak hip adduction angle, were investigated with 3D motion analysis during walking. The study hypothesis was rejected as no hip abductor muscle weakness was determined in the children with increased femoral anteversion. According to the kinetic analysis results, there was no significant difference found in the peak hip abductor moment (*p* = 0.35) compared to the control subjects. This result is consistent with the literature that has investigated the hip abductor moment in adolescents with increased femoral anteversion [27]. In order to rule out potential causes of muscle weakness, the study only included participants who did not have generalized joint hypermobility. The Beighton score is one of the reliable methods used to determine generalized joint hypermobility [17]. There is information in the literature that states that generalized joint hypermobility affects joint stability by reducing muscle strength [28,29]. Therefore, there are no possible effects of generalized joint hypermobility on the hip abductor maximum voluntary isometric contraction measured in this study.

In our study, a portable handheld dynamometer was used to measure hip abductor muscle strength in the side-lying position. Intra-rater ICC was reported to be high for hip abductor isometric muscle strength measured with a portable handheld dynamometer [30]. Muscle strength measured with a dynamometer can vary depending on various factors, such as body size, gender, and age. Body size is one of the well-known factors influencing the outcome of a muscle strength test. Although the normalization of muscle strength values measured by a dynamometer according to body size is conducted with different formulas, there is no consensus regarding the superiority of these methods over each other [31]. In this study, the muscle strength values (Newtons) of the participants measured with a dynamometer were normalized according to their body weights (Newton/Kg). Accordingly, there was no significant difference in the hip abductor muscle strength of the participants with increased femoral anteversion compared to the controls. Arnold et al. (1997), in their study with computer modeling, stated that increased femoral anteversion reduced the abductor moment of the gluteus medius muscle [10]. This finding was one of the first publications in the literature stating that femoral anteversion may cause hip abductor weakness. In another study, the gluteus medius muscle EMG amplitudes of those with increased femoral anteversion were found to be 34% lower than those of control subjects [11]. Nylan et al. (2004) stated, with EMG data, that increased femoral anteversion causes gluteus medius muscle weakness, but also that the effects on walking were not examined [11]. In the current study, consistent with the undiminished hip abductor moment, there was also no increase in the peak hip adduction angle observed during the stance phase. A possible abductor muscle weakness may lead to an increase in the hip adduction angle in the stance phase, as seen in the Trendelenburg gait. In another study with a small number of participants, there was reported to be no increase in the kinematics of hip adduction and contralateral pelvic drop when the isometric hip abductor strength (maximum voluntary contraction (N m kg^−1^)) was reduced by 46% to 26% by applying a superior gluteal nerve block [23]. There may be compensatory strategies for maintaining the normality of hip joint frontal plane mechanics in such hip abductor muscle weakness. This compensation can be made by the ipsilateral trunk lean during the stance phase [32]. In this study, trunk movements were not investigated.

In an analysis of running at 4 m s^−1^ speed, it was stated that there was no relationship between isokinetic hip abductor muscle strength (N m kg^−1^) and frontal plane hip joint kinematics. However, it was reported that there is a relationship between the femoral anteversion angle and the hip adduction angle (*r* = 0.41) [16]. According to the results of the gait analysis performed in the current study, at an average speed of 1.13 m/s there was no correlation found between the femoral anteversion and the peak hip adduction angle in the stance phase (*r* = 0.149, *p* = 0.49). An increase in femoral anteversion has been shown to lead to an increase in the hip adduction angle in the frontal plane while running, but not in walking. However, in the current study, the hip adduction angles of the children with increased femoral anteversion were not statistically different from those of the control group (*p* = 0.08). The results of this study showed that increased femoral anteversion was not associated with hip abductor muscle weakness and increased hip adduction in walking. Passmore et al. (2018) reported that hip adduction angles increased in participants with increased femoral anteversion. However, the hip adduction angle in the stance phase alone was not specified, and it was stated that those with increased femoral anteversion walked more slowly than the controls [27]. It is known that walking speed is one of the factors that can directly affect the kinematic parameters of walking. Increased hip adduction in the stance is associated with many lower extremity alignment problems [13]. The results of this study showed that increased femoral anteversion did not increase hip adduction in the stance phase of walking. Increased femoral anteversion is characterized by an increased hip internal rotation in walking. Arnold et al. (1997) stated that the increase in the hip internal rotation in walking developed as compensation for hip abductor weakness in increased femoral anteversion, although other muscles also contribute to hip abduction during walking [10]. In the current study, peak hip internal rotation angles in the stance phase were higher in participants with increased femoral anteversion compared to the control group (*p* = 0.003). It can be considered that the increased pelvic rotation angle may be part of the compensation mechanism in addition to hip rotation. Apart from derotation osteotomy, treatment methods that force the hip joint to external rotation may cause hip abductor weakness. However, the literature is limited on the conservative treatments of increased femoral anteversion. Surgical derotation osteotomy is generally applied to cases with neurodevelopmental disorders such as cerebral palsy. In the study of Boyer et al. (2017), it was stated that there was a significant increase in hip abductor moments and isometric hip abductor muscle strength in children with cerebral palsy who underwent derotation osteotomy in the postoperative three-year period [33].

Studies investigating the increase in femoral anteversion in the literature have determined anteversion in two different ways: radiological methods (e.g., MRI, CT, and ultrasonography) and clinical methods (maximal hip internal and external rotation range of motions, and Craig’s test). Craig’s test was reported to have a higher correlation with radiological methods than the maximal hip internal rotation angle [34]. The study by Ruwe et al. found that the Craig’s test method had a strong correlation with intraoperative measurements and radiological measurements [18]. It was noted that there was excellent concurrent validity (*R* = 0.862, *p* < 0.001) between femoral anteversion measurements made with Craig’s test and the two-dimensional computed tomography method in the study by Chung et al. (2010) on patients with cerebral palsy [34]. However, information about the correlation of clinical methods with radiological methods in the literature is not compatible with each other [35]. Although there is disagreement in the literature regarding the accuracy of clinical measurement techniques for measuring femoral anteversion angles, this disagreement is not present for radiological techniques. The limitation of this study is that the increased femoral anteversion was not measured by radiological methods. In this study, while determining the participants to be included in the increased femoral anteversion group, in addition to the ≥30 degrees Craig’s test, at least 40 degrees difference between the hip internal rotation angle and the external rotation angle was applied. Considering these criteria, the statistically significant difference in the femoral anteversion angles of the participants in the two different groups is thought to adequately reflect the effects of increased femoral anteversion in all our findings.

Increased femoral anteversion does not cause hip abductor muscle weakness. Ipsilateral hip adduction and contralateral pelvic drop angles are not increasing with increased femoral anteversion at the stance phase of the participants’ self-selected walking speed. Increased femoral anteversion affects the transverse plane hip joint biomechanics rather than the frontal plane. According to the results of this research, strengthening hip abductor muscles may not be beneficial for children with increased femoral anteversion. The relationship between musculoskeletal problems associated with hip abductor muscle weakness and increased femoral anteversion should be investigated in more detail. Further research is needed to determine how the reduction in or restriction of increased lower extremity internal rotation due to increased femoral anteversion will affect pelvis and hip frontal plane kinematics.

## Figures and Tables

**Table 1:** Demographics of the participants, including the hip rotation angles of participants measured by goniometer and hip abductor MIVC values.

	Control *n* = 22 Mean ± SD	IFA *n* = 18 Mean ± SD	*p*-Value
Age (year)	10.25 ± 3.98	9.5 ± 2.97	0.385
BMI (Kg/m^2^)	18.10 ± 6.05	18.49 ± 3.5	0.461
Maximum hip internal rotation (°)	38.95 ± 13.66	68.33 ± 6.34	0.001 *
Maximum hip external rotation (°)	31.55 ± 7.52	18.5 ± 4.21	0.003 *
Craig’s test (°)	21.25 ± 12.25	42.83 ± 10.29	0.003 *
Normalized hip abductor MIVC (Newton/kg)	3 ± 0.89	4 ± 0.57	0.14

BMI, body mass index; MIVC, maximal isometric voluntary contraction; and IFA, increased femoral anteversion, *p* < 0.05 *.

**Table 2:** Gait analysis results for the pelvis and lower body kinematics.

	Control *n* = 22	IFA *n* = 18	*p*-Value
Gait velocity (m/s.)	1.27 ± 0.11	1.13 ± 0.13	0.96
Pelvic obliquity ROM@%GC (°)	6.33 ± 2.55	9.67 ± 5.36	0.025 *
Peak pelvic depression@%ST (°)	−2.8 ± 1.92	−4.25 ± 3.67	0.149
Peak pelvic rotation@%GC (°)	8.76 ± 4.56	14.12 ± 5.77	0.022 *
Peak pelvic tilt@%GC (°)	11.44 ± 2.78	12.82 ± 5.89	0.371
Peak hip adduction@ST (°)	4.34 ± 4.03	6.12 ± 4.97	0.088
Peak hip internal rotation@ST (°)	3.84 ± 5.31	12.24 ± 7.32	0.003 *
Peak hip extension@ST (°)	−13.01 ± 5.42	−8.71 ± 6.78	0.085
Peak foot internal rotation@ST (°)	−9 ± 7.59	−3.18 ± 7.29	0.022 *
Hip adductor moment@ST (N*m/kg)	0.20 ± 0.15	−0.32 ± 0.33	0.35

GC, gait cycle; ST, stance; ROM, range of motion; IFA, increased femoral anteversion; (°), angle; N*m/kg, Newton*m/kg; and m/s, meter per second, *p* < 0.05 *.

## Data Availability

The datasets used and/or analyzed during the current study are available from the corresponding author on reasonable request.
